# Geo-Economic Linkages between China and the Countries along the 21st-Century Maritime Silk Road and Their Types

**DOI:** 10.3390/ijerph191912946

**Published:** 2022-10-10

**Authors:** Wei Hu, Yuejing Ge, Zhiding Hu, Shuai Ye, Feng Yang, Haining Jiang, Kun Hou, Yun Deng

**Affiliations:** 1 College of Geography and Environmental Sciences, Zhejiang Normal University, Jinhua 321004, China; 2 Faculty of Geographical Science, Beijing Normal University, Beijing 100875, China; 3 Institute for Global Innovation and Development, East China Normal University, Shanghai 200062, China; 4 School of Geographical Science, Qinghai Normal University, Xining 810008, China; 5 School of Environmental Science and Engineering, Southern University of Science and Technology, Shenzhen 518055, China; 6 School of Remote Sensing and Geomatics Engineering, Nanjing University of Information Science and Technology, Nanjing 210044, China

**Keywords:** geo-economic linkage, geo-economic types, extreme random forest regression model, China, the 21st-Century Maritime Silk Road (MSRCs)

## Abstract

With the advances of the Belt and Road Initiative (BRI), the geo-economic interactions between China and countries along the 21st-Century Maritime Silk Road counties (MSRCs) continue to increase, and analyzing the geo-economic relations between China and the MSRCs is extremely important for a scientific understanding of bilateral geo-economic cooperation. Differently from the traditional logic of geo-economic competition and cooperation, we constructed a new framework based on the dominant factors of geo-economic relations and used an extreme random forest regression model to classify the geo-economic relation types between China and MSRCs from 2006 to 2017. The results show that the unbalanced development of investment and trade between China and MSRCs hindered the enhancement of the intensity of bilateral geo-economic linkage from 2006 to 2017. The “Matthew effect” of China’s geo-economic flow linkage with MSRCs is significant. There are obvious differences in the dominant factors affecting the types of geo-economic relations between China and MSRCs, and the distribution of the importance of the indices of the types of geo-economic relations in each country is disordered. Geopolitics, markets, and resources have played important roles in the geo-economic linkages between China and MSRCs. There are five types of geo-economic relations between China and the MSRCs: market-oriented type, resource-oriented type, market-resource-oriented type, market-geopolitics-oriented type, and resource-geopolitics-oriented type, of which the market-oriented type is the most important type of geo-economic relations. In the future, China should focus on regional powers along the Maritime Silk Road for bilateral geo-economic cooperation, actively promote the balanced development of bilateral geo-economic elements flows, strengthen geopolitical cooperation with MSRCs, and formulate cooperation plans according to the types of geo-economic relations.

## 1. Introduction

In the geo-economic era of globalization, the interactions and transformation of geo-economic factors are more rapid, geo-economic networks have become larger and more complex, and geo-economic interactions between countries are more complex than ever before [1]. As the Belt and Road Initiative (BRI) continues to be promoted, 205 cooperation documents to jointly build the Belt and Road have been signed between China and 171 countries and international organizations [2], and China’s circle of friends for the BRI is continuously expanding. In this context, the geo-economic linkages between China and MSRCs have been further strengthened. In 2020, China’s total imports and exports of goods with MSRCs reached 1262.4 billion dollars. Among MSRCs, Singapore, Indonesia, Vietnam, Laos, Malaysia, and Saudi Arabia play important roles in Chinese enterprises’ overseas investment and contracting projects. As major shipping channels, trade channels, and energy channels for China, MSRCs are the regions where China’s geo-economic interests are primary and are the key areas for China to expand its geo-economic space.

Through the 21st-Century Maritime Silk Road, China is engaged in broader, higher-level, and deeper regional cooperation, and is working to build a mutually open, inclusive, balanced, and beneficial geo-economic cooperation architecture [3]. In the process, new free trade agreements were signed between China and many other countries [4], infrastructure connectivity between China and Asia, Europe, and Africa has been strengthened and improved [5], links have been established between the dynamic East Asian economic zone and the developed European economic circle, and the geo-economic structure of China and MSRCs is changing [6]. On the one hand, the flow of geo-economic linkage factors such as trade flows, investment flows, and tourism flows between China and the MSRCs has reached a new height; China’s central position in the geo-economic network of MSRCs is increasing. On the other hand, the “space of flow” structure shaped by the geo-economic linkage elements flow between China and MSRCs is being upgraded; geo-economic “space of place” is gradually replaced by “space of flow.” However, although there has been research on the geo-economic intention, geo-economic network, and geo-economic effects of the 21st-century Maritime Silk Road [7], these studies mainly focused on the geo-economic competition faced by the 21st-century Maritime Silk Road, though less attention has been paid to the evolution of the characteristics of geo-economic linkages between China and MSRCs, and the characteristics of geo-economic relations between China and MSRCs remain unclear.

To scientifically reveal the changes in the geo-economic linkages and their types between China and MSRCs, we constructed a new analytical framework of geo-economic relations. Firstly, this study takes MSRCs as the research object and constructs the geo-economic flow linkage intensity model based on flow space theory to analyze the evolution of the characteristics of geo-economic linkages between China and MSRCs. Then, an index system of geo-economic relation types is constructed, and the extreme random forest regression model is used to identify the importance of the geo-economic relation type indices and determine the dominant factors of geo-economic relations between China and MSRCs. Finally, this research divides the geo-economic relations between China and MSRCs into different types based on the importance of the geo-economic relation type indices.

The design of this study is as follows. In Section 1, the important influence of the construction of the 21st-Century Maritime Silk Road on the geo-economic linkages between China and MSRCs is introduced. In Section 2, the related research literature is reviewed, and the linkages and differences between this research and existing research are detailed. In Section 3, the research method, research area, and research data are introduced. In Section 4, the evolution characteristics of the geo-economic linkage between China and MSRCs are analyzed, the dominant factors of the geo-economic linkage between China and MSRCs are identified, and the geo-economic relation types between China and MSRCs are classified. In the last section, the conclusions and policy implications are presented.

## 2. Literature Review

Since its birth, the concept of geo-economy has had a strong geopolitical color. When Luttwak proposed the concept of the geo-economy, he emphasized that economic power and economic means are important aspects of the game between powerful countries and the distribution of power [8]. Blackwill and Harris understood geo-economics as the use of economic means to achieve political goals [9]. Vihma stated that the definition of geo-economics is very broad, covering a number of matters, such as cross-border economic zones, foreign policy, neoliberalism, and economic nationalism [10]. Hudson defined geo-economics as a strategy for regional development through economic means, such as trade and investment [11]. Baru pointed out that as geo-economics is the interaction between the economy and geopolitics, economic development has geopolitical influence, and geopolitical changes will affect the economy [12]. Although geo-economics is indeed affected by geopolitics, Western economics considers that it is not appropriate to completely limit geo-economics to the scope of geopolitics [13]. New mercantilism believes that geo-economics aims to achieve economic wealth rather than political benefits [14]. Neoliberal institutionalism says that geo-economic strategic practice is the country’s participation in regional economic cooperation as a rational economic man, and the common interest is the key to cooperation [15]. New trade theory believes that a large-scale economy guides the country to participate in the division of labor and specialization; and international trade, resource differences, scale benefits, and agglomeration benefits shape the global trade structure [16]. In fact, geo-economics represents kinds of economic relationship formed on the basis of geographical location, resource endowment, and economic structure, such as cooperation, complementarity, competition, and opposition [17]. The regional potential difference caused by the spatial differentiation of geo-economic elements promotes the global flow of geo-economic elements, resulting in various geo-economic flows, such as trade, investment, and tourism flows. According to the space of flow theory, socio-economic space is the collection of “flow” of socio-economic elements [18], and the “flow” of socio-economic elements shapes the socio-economic space. With the support of modern information technology and transportation technology, flows of geo-economic elements between countries are becoming increasingly rapid, and not only maintain geo-economic ties between countries, but also shape the “flow space” of geo-economic ties. Therefore, geo-economic element flows are the basis of geo-economic relations, and when analyzing geo-economic relations, it is necessary to pay attention to geo-economic element flows among countries.

Geo-economic competition and cooperation, and geo-economic relations matching are the key topics of geo-economic linkage research. Geo-economic competition and cooperation mainly focus on the competitiveness, cooperation, and complementarity of geo-economic relations. The geo-economic flow between China and the countries along the Belt and Road is increasing, and the geo-economic relations between China and these countries are continuing to improve [19]. After China’s accession to the WTO, Taiwan’s dependence on China’s exports has increased by three percentage points, which will further deepen the economic dependence between the mainland and Taiwan [20]. The matching of geo-economic relations focuses on the types and patterns of geo-economic relations between countries or regions. Udeala considered the main south–south geo-economic cooperation model asymmetric, and the economic relations between Nigeria and China very unbalanced [21]. Wong and Chan argued that China and ASEAN countries have certain similarities in trade structures and industrial structures, and that the two sides are more competitive than complementary [22]. Geo-economic linkages are relatively stable in the short term, but will develop with the evolution of various geo-economic elements in the long term. With the continuous growth of China’s investment and trade in North Korea, the relations between China and North Korea have shifted from China’s unilateral economic support to strategic cooperation [23]. In the process of economic globalization, China’s geo-economic influence has gradually expanded from China’s periphery to the world [24]. Japan and Russia are highly complementary in the automotive and energy industries, and the geo-economic relations between the two countries have improved with the eastward movement of the Russian economy [25]. China has fairly good trade and investment relations with all its trading partners, but almost all of China’s major trading partners have imposed trade restrictions on China’s exports, which has seriously affected the development of China’s foreign economic relations [26]. China’s economic reforms have made China’s economic position more stable than before, but at the same time may intensify trade tensions between China and the United States, and may even cause the United States to impose trade sanctions [27]. It is worth noting that quantitative research methods such as social network analysis, regression analysis, and Bayesian alternative finding are constantly enriching geo-economic research [28]. Maoz introduced social network analysis methods into the field of international relations research, and visualization revealed the network characteristics of the international relations from 1816 to 2001 [29]. Strüver used logit regression to test the impacts of ideology and economic interests on China’s partnership diplomacy, and highlighted the importance of economic interests in explaining partnership onset [30]. Pang proposed a Bayesian alternative to the synthetic control method for comparative case studies with a single or multiple treated units, which has a good application in determining the economic impacts of German reunification, election day registration, and voter turnout [31]. Getmansky used difference-in-difference regressions to estimate the effect of barrier construction on automobile theft, and border fortification may have uneven distributional consequences and create unintended winners and losers [32]. Chen use gravity equation to estimate border effects among European Union countries, and find technical barriers and product-specific information costs increase border effects [33]. Unfortunately, these quantitative research methods are helpful to reveal the complexity of geo-economic interaction among countries, but they cannot effectively identify the type characteristics of geo-economic relations.

Geo-economic elements play an important role in the changing of geo-economic linkages. Geo-economic factors such as resources, trade, finance, and technology are shaping the competition and cooperation of today’s geo-economy [34], as resource conflicts are geo-economic and geopolitical conflicts [35]. Joint investment and cooperation in raw materials and minerals between developed and developing countries can reduce competition between them [36]. Trade agreements have a great impact on regional trade and investment relations, and may reshape the geo-economic linkage between countries [37]. The expansion of trade scale and trade flow is positively correlated with the geo-economic linkages between countries [19]; and trade barriers, export restrictions, and trade wars will cause declines or even interruptions in geo-economic linkages [38]. The rising position of finance in geo-economic cooperation, the provision of liquidity support, development financing, and the internationalization of the RMB are making China’s financial ties with Asian countries closer [39]. Technological advantages can become the basis of power, and the technological advantages of the United States give it a favorable position in geo-economic competition [40]. In addition, new environmental variables such as disasters, refugees, and climate change have an increasing impact on geo-economic linkages [41].

The ways in which various influencing factors act on geo-economic linkages extend from resource and political tools to technical tools [42]. Different influencing factors bring about different paths to shape geo-economic linkages, such as the geo-environment, expansion of multinational companies, and geo-economic competition [43]. A variety of influencing factors and influencing paths jointly promote the evolution of geo-economic linkages, the mechanism of whose influence varies from region to region. Geo-economic linkages between countries and regions depend largely on geopolitical linkages [42]. China is regarded by the EU as a challenging economic power, a geopolitical suspicion that adds tension to the development of geo-economic relations between China and Europe [44]. The market is the most important driving force for geo-economic ties between China and South American countries [24]. In addition, driving factors such as geo-location, geo-cultural factors, and national policy also play important roles in the mechanisms of influence of geo-economic linkages [45].

In the process of constructing the BRI, the flow of geo-economic elements such as trade, transportation, and resources is being drawn into a new map of geo-economic linkages among the MSRCs [46]. In terms of trade, the BRI is expected to promote the signing of free trade agreements between China and other countries in the world, reduce tariffs, and promote geo-economic cooperation [4]. Regarding infrastructure connectivity, the BRI has invested heavily in infrastructure such as roads, railways, ports, and pipelines to strengthen and improve connectivity between China and countries along the Maritime Silk Road [47]. In terms of investment, Belt and Road construction has made the Maritime Silk Road a priority area for China’s investment [7], significantly promoting the investment growth of countries along the Maritime Silk Road [48]. At the market level, the 21st-Century Maritime Silk Road was built to promote the free flow of economic factors and create a unified regional market and industrial value chain [6]. This constitutes the geo-economic effect of the construction of the 21st-Century Maritime Silk Road. The importance of China’s position in the global economic landscape has increased substantially [43], and the increasing participation of Chinese enterprises in the management of overseas ports has led to the formation of a shipping network with China as its core [49].

There is no doubt that the perspectives and content of geo-economic research are becoming increasingly diversified, and geo-economic elements such as resources, trade, finance, and technology play key roles in shaping geo-economic linkages. The construction of the Belt and Road has created closer and more frequent geo-economic exchanges between China and MSRCs than before, and bilateral geo-economic linkages are also showing new characteristics. However, the existing studies are limited to the logic of geo-economic competition and cooperation when analyzing the geo-economic relations between China and MSRCs, and have not clarified the dominant factors affecting the evolution of geo-economic linkage between China and MSRCs, nor do they propose a method to classify the types of geo-economic relations between China and MSRCs. Therefore, this study addresses the following three questions: (1) How do the geo-economic linkages between China and MSRCs evolve? (2) What factors drive the evolution of geo-economic relations between China and MSRCs? (3) How can one classify the types of geo-economic relations between China and MSRCs? To solve these problems, we constructed a new analytical framework to analyze the characteristics of the geo-economic linkages and geo-economic relation types between China and MSRCs based on geo-economic element flow.

## 3. Methods and Data

### 3.1. Geo-Economic Flow Linkage Intensity Mode

The flow space theory of Castells pointed out that the dominant form of space is no longer the local space in the network society, but a new flow space [18]. In the process of globalization, the spatial structure of the global economy is being reshaped by the flow network of trade flows, investment flows, resource flows, information flows, and technology flows. The dependence of geo-economic development on “location space” has gradually turned to “flow space” [19]. Geo-economic flows have become the key to maintaining and driving geo-economic relations. It is the flow of resources, trade, investment, tourism, information, and other geo-economic elements that is affected by geographic distance, geo-economic space, and geopolitical cooperation among different geo-entities. As the core variable of a geo-economic relation, geo-economic flow shapes the geo-economic linkages, and geo-economic flow is the reflection of geo-economic linkages. Although geo-economic flows have significant geo-relation attributes, geo-economic flows are not the entirety of geo-economic relations. In contrast to international economic relations, geo-economic relations are the weightings of economic relationship by geo-variables. Geo-variables caused by environmental differences such as geopolitics, geo-culture, and geo-traffic are endogenous to geo-economic. Therefore, geo-economic relations should be analyzed in consideration of geo-economic flows and geo-variables.

In related studies, geo-economic linkage intensity is often measured using gravity models and gray correlation analysis [50]. However, these studies emphasize the economic variables in geo-economic relations; the interaction of geo-economic elements’ flow does not receive attention, geo-variables are not integrated, and the “flow space” characteristics of geo-economic relations cannot be identified. Referring to Keohane’s interdependence theory and Taylor’s flow space network research method [15,51], we used the square root of the product of the geo-economic linkage elements’ flows multiplied by the geo-variables to represent geo-economic linkage intensity and construct a geo-economic flow linkage intensity model. Owing to the numerous elements of geo-economic flows, it is impossible to incorporate all the elements of geo-economic flows into the geo-economic linkage intensity model in quantitative statistics. Trade and investment flows are the core elements of geo-economic flows, which can cover the main aspects of geo-economic linkages when analyzing the intensity of geo-economic flow linkages. Geo-culture is used more as a geopolitical tool in international exchanges, and its impact on the geo-economy is weaker than those on geopolitics and geo-traffic. The overall geo-cultural conflict of the MSRCs is relatively gentle, and geo-culture has a limited effect on China’s geo-economic interactions with the MSRCs. Therefore, geo-variables mainly consider geopolitics and geo-traffic. China’s economic and trade exchanges with the MSRCs mainly rely on maritime transport, and the bilateral transport links are mainly maritime transport links. Therefore, this study considers trade and investment as the elements of geo-economic flow, and takes the goldenstein factor that reflects geopolitical cooperation and conflict and the liner shipping connectivity index that reflects the strength of marine transportation connectivity as the geo-variables. The specific geo-economic flow linkage intensity model is as follows:

(1) $$L_{i}=\sum_{j=1}^{n}L_{ij}$$

(2) $$L_{ij}=(\sqrt{T_{i}\cdot T_{j}}+\sqrt{I_{i}\cdot I_{j}})*(rp+rd)$$

where *L_ij_* is the geo-economic flow linkage intensity between *i* and *j*, *T_i_* is the trade flow from *i* to *j*, *T_j_* is the trade flow from *j* to *i*, *I_i_* is the investment flow from *i* to *j*, *I_j_* is the investment flow from *j* to *i*, *rp* is the political geopolitical variable, *rd* is the transportation geopolitical variable, and *L_i_* is the total geo-economic flow linkage intensity in *i*.

The geo-economic flow linkage intensity reflects the closeness of geo-economic relations among countries. The greater the value of *L_ij_*, the closer the geo-economic relations. The geo-economic flow linkage intensity model objectively evaluates the strengths and weakness of geo-economic interactions among countries based on geo-economic element flow. It covers the most important investment flows and trade flows in geo-economic linkages. It can not only reveal the vector characteristics of geo-economic flows between countries, but can also reveal the evolution characteristics of geo-economic relations.

### 3.2. Extreme Random Forest Regression

The random forest is an integrated algorithm based on the decision tree proposed by Breiman [52], which is mainly used for classification and regression problems. In random forests, the dependent variable can be either categorical or continuous. It does not require a large number of parameter adjustments and has a high classification accuracy and low risk of overfitting. It can effectively measure the importance of an eigenvalue in the classification and can evaluate and rank the importance of explanatory variables [53]. Random forest regression is one of the most important uses of the RF algorithm. Random forest regression does not require a functional form, the dataset does not need to be standardized, the regression or classification results are not affected by the lack of individual data, and there is no requirement for the correlation of explanatory variables [52]. The regression tree can be constructed with a small number of variables, which can better explain the nonlinear relations between the explained variables and explanatory variables. An extremely randomized tree is a special random forest tree. Each decision tree of an extremely randomized tree applies the same training sample. It randomly selects one of the best splitting attributes to split, which is faster than ordinary random forest training. More importantly, extreme random forest regression combines the advantages of random forest regression and the extremely randomized tree, which can overcome the limitations of possible autocorrelation among geo-economic relation type indices and missing individual data, and can also quickly measure the importance of geo-economic relation type indices of MSRCs. Therefore, we used Python to conduct extreme random forest regression on geo-economic relation type indices to obtain the importance of geo-economic relation type indices of MSRCs. The importance of the characteristic variable of each random forest is indicated by a positive value ranging from 0 to 1. The importance of a characteristic variable indicates the importance of the characteristic variable’s contribution to the explained variable, and the importance of all characteristic variables adds up to 1. To test the effect of the random forest regression model, the R-square (R^2^), mean square error (MSE), mean absolute error (MAE), and explained variance score (EVS) are used to test the effect of random forest regression estimation.

### 3.3. Research Area and Data Sources

**Figure 1:**
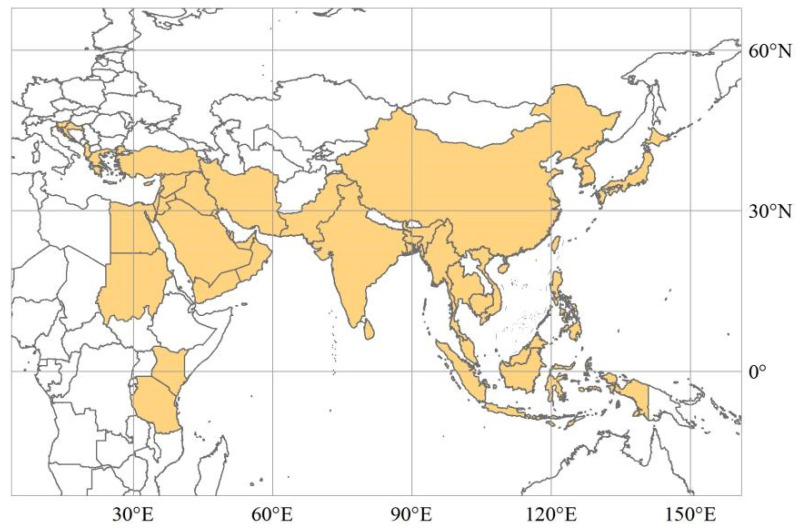
Study area. Note: The map is a drawing is based on the standard map of GS (2016) 1667 that come from the standard map service system of the Ministry of Natural Resources of the People’s Republic of China (http://bzdt.ch.mnr.gov.cn/index.html, accessed on 3 October 2022).

The MSRCs have no precise spatial scope as part of the BRI [3]. Historically, the Maritime Silk Road has mainly included the East China Sea shipping routes, South China Sea shipping routes, and North American and South American shipping routes. Since the vast majority of American countries have not signed a Belt and Road cooperation agreement or memorandum with China, this study focuses on the East China Sea shipping route countries and South China Sea shipping route countries. Combined with the ancient Maritime Silk Road route and reference data availability, we selected 39 MSRCs that are shown in Figure 1 as the study area. To facilitate the analysis, the 39 MSRCs besides China were divided into three East Asian countries, nine Southeast Asian countries, four South Asian countries, fourteen West Asian countries, five Balkan countries, and four African countries (Table 1). The research data mainly came from the UN Comtrade database, World Bank data, CEPII databases, GDELT Event Database, China Statistical Yearbook, World Investment Report, China foreign investment statistics bulletin, Statistics Bureau of the People’s Republic of China, the website of the Ministry of Foreign Affairs of the People’s Republic of China, the website of the Ministry of Commerce of the People’s Republic of China, the website of the National Bureau of Statistics of the People’s Republic of China and the website of Wuliu Baba. Although the trade data between China and MSRCs from 2006 to 2020 can be obtained, there is a lack of investment data between China and MSRCs after 2017, which makes it impossible to measure the linkage intensity between China and MSRCs and the characteristic importance of geo-economic relation type indices after 2017. Therefore, this study analyzes geo-economic linkages and their types between China and MSRCs from 2006 to 2017.

## 4. Results

### 4.1. Analysis of Geo-Economic Linkage Intensity between China and MSRCs

#### 4.1.1. The Unbalanced Development of Investment and Trade between China and MSRCs Hinders the Enhancement of the Intensity of Bilateral Geo-Economic Linkages

**Figure 2:**
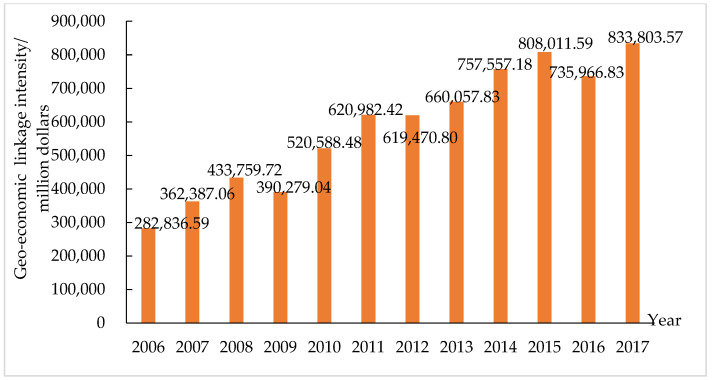
The variation of geo-economic linkage intensity between China and the MSRCs.

The intensity of the geo-economic linkages between China and MSRCs was measured by the geo-economic flow linkage intensity model, and it was found that the intensity of geo-economic linkages between China and the MSRCs rose from 282,836.59 million dollars in 2006 to 833,803.57 million dollars in 2017 (Figure 2). This shows that the geo-economic ties of China and the MSRCs continue to strengthen, further enhancing bilateral geo-economic relations. According to the growth characteristics of the intensity of geo-economic linkages, the changes in the geo-economic relations between China and the MSRCs can be divided into the initial period of development (2006–2008), the period of rapid growth (2009–2013), and the period of consolidation and strengthening (2014–2017). In the early stage of development, China was in a period of rapid economic growth, foreign investment and foreign trade exports continued to rise, the intensity of geo-economic flow ties rose to 433,759.72 million dollars, and bilateral geo-economic relations achieved rapid development. In the period of rapid growth, China’s economy was shifting from high speed to medium-low speed growth. Its exports continued to grow, and outbound investments increased. During this period, the average annual growth rates of total trade flows and investment flows between China and the MSRCs reached 16.15% and 17.40%, respectively, which led to a rapid increase in the intensity of bilateral geo-economic flows, reaching 660,057.83 million dollars in 2013. In the period of consolidation and strengthening, China has actively promoted the construction of the Belt and Road, and the MSRCs have become key regions of China’s geo-economic development—further strengthening bilateral economic and trade cooperation, increasing China’s investment in the regions along the routes, and enhancing the linkage intensity of geo-economic flows.

The geo-economic relations between China and the MSRCs have been greatly impacted by the financial crisis, global economic adjustments, and recovery of trade protectionism. Due to these factors, the geo-economic flow between China and the MSRCs fell in 2008, 2012, and 2016; the geo-economic competition intensified; and the geo-economic linkage intensity decreased. Since the construction of the BRI, the intensity of China’s geo-economic linkage with the MSRCs has not increased rapidly, but it has increased in volatility. On the one hand, under the influence of the global trade situation and economic growth, China’s trade and investment relations with the MSRCs have increased in volatility. On the other hand, the development of investment and trade between China and the MSRCs is unbalanced. China’s investment in the MSRCs is far greater than China’s investments in China, and China’s exports to the MSRCs are far greater than their exports to China. This imbalance hinders the improvement of the intensity of the geo-economic linkages between China and the MSRCs to a certain extent.

#### 4.1.2. The “Matthew Effect” of China’s Geo-Economic Linkage with MSRCs Is Significant

**Figure 3:**
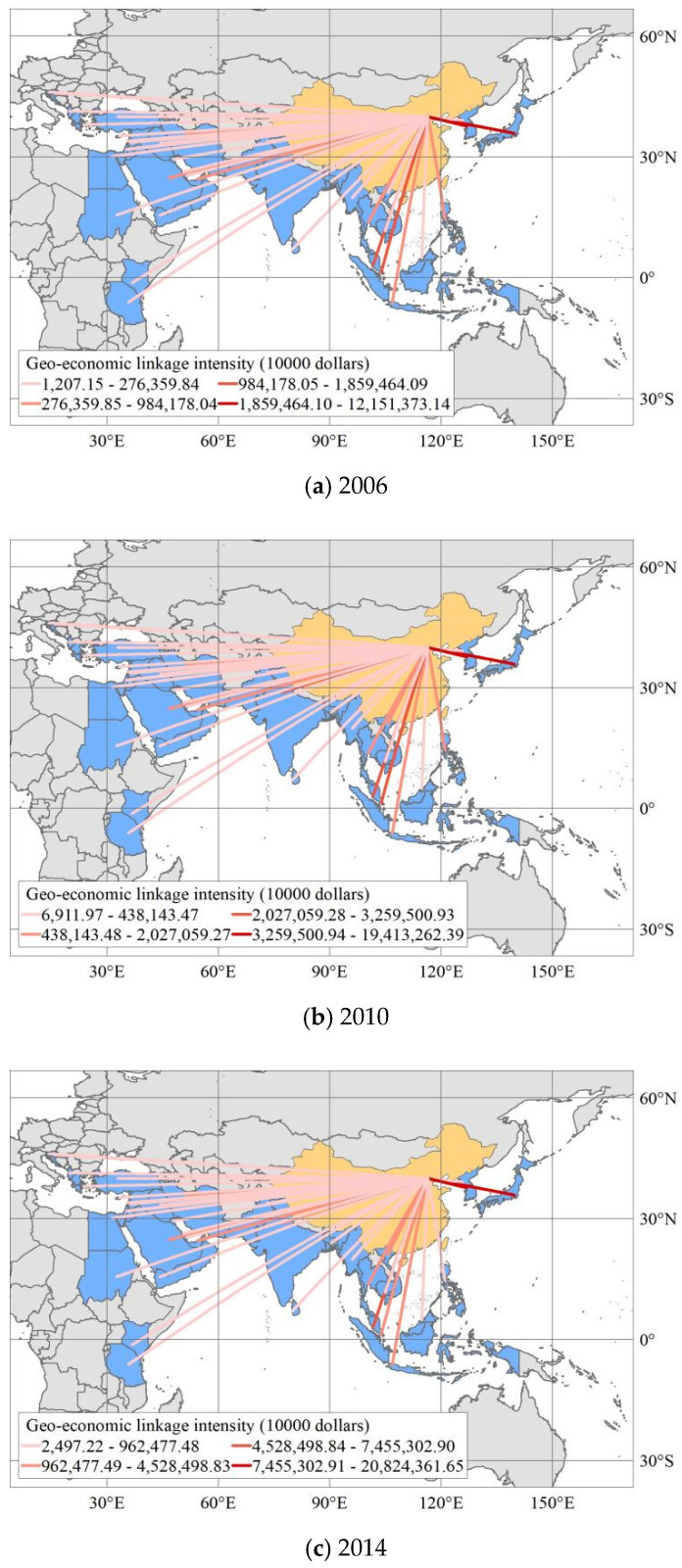

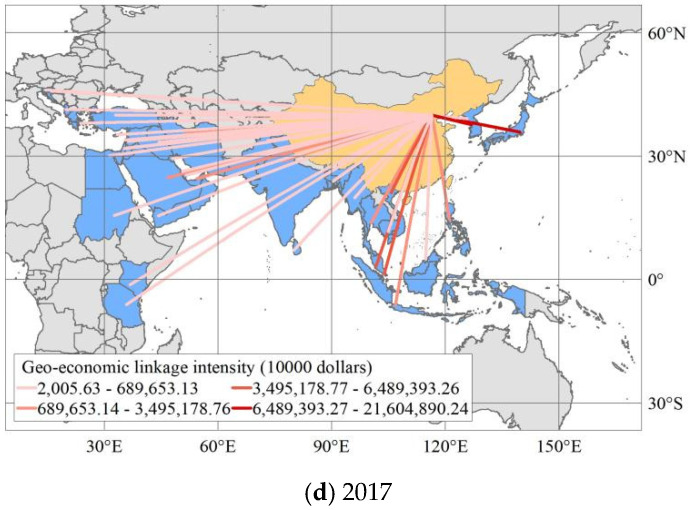
The spatial distribution of geo-economic linkage intensity between China and MSRCs. Note: The map is a drawing is based on the standard map of GS (2016) 1667 that comes from the standard map service system of the ministry of natural resources of the People’s Republic of China (http://bzdt.ch.mnr.gov.cn/index.html, accessed on 3 October 2022).

In order to reveal the evolution of geo-economic linkage intensity between China and MSRCs, we selected 2006, 2010, 2014, and 2017 as time nodes and used ArcGIS software to visualize them (Figure 3). According to the spatial distribution of the geo-economic linkage intensity of the geo-economic flows between China and the MSRCs, it was found that the linkage intensities of geo-economic flows between China and Japan and South Korea are the highest; and those between China and Singapore, Malaysia, and Vietnam are relatively high. The intensity of the geo-economic linkages between China and Thailand, India, the Philippines, Saudi Arabia, Indonesia, the United Arab Emirates, and Iran are low; and the intensity of the geo-economic linkages between China and other countries is the lowest. From the perspective of the proportion of the number of countries with different linkage intensity levels, the linkage intensity between the geo-economic flows of China and most of the MSRCs is low. Taking 2017 as an example, there are 27 countries with low linkage intensity, and low and low linkage intensities are dominant. From the perspective of regional division, the countries with low geo-economic linkage with China include five Balkan countries, four African countries, and the Middle East. These countries are not preferred countries for China’s overseas investment because of their relatively small economies, small import and export volumes with China, and high transportation costs for bilateral geo-economic activities due to the long distance.

Countries with high geo-economic linkages with China are concentrated in Southeast Asia, Japan, and South Korea. These countries are surrounded by China, Japan, and South Korea, which have large economic volumes, broad ASEAN markets, strong geo-economic complementarity, and frequent bilateral trade and investment exchanges. In addition, bilateral geo-economic growth has been sustained by the construction of the China–ASEAN free trade area and the China–South Korea free trade area. Over time, the pattern of geo-economic relations between China and the five Balkan Peninsula countries, four African countries, and fourteen West Asian countries has been relatively stable; and the pattern of geo-economic relations between China, Southeast Asia, and South Asia has changed greatly. From 2006 to 2010, the geo-economic connection intensity between India and China rose from a low to a high level. Since then, the growth rate of the strength of Sino-Indian geo-economic ties has been slow, and the strength of bilateral ties has declined, being affected by factors such as different development stages, trade imbalances, and geopolitics. According to the intensity ranking of geo-economic linkages, the intensity of geo-economic linkages between China and Vietnam increased the fastest, and that between China and Syria declined the fastest. From 2006 to 2017, Vietnam rose from 12th to 3rd, and Syria dropped from 28th to 39th. The industrial structure and resource endowments of China and Vietnam are highly complementary; a large number of Chinese enterprises invest in factories in neighboring Vietnam. Bilateral trade flows and investment flows have increased rapidly, from 2748.04 million dollars in 2006 to 64,837.86 million dollars in 2017, the largest increase in all countries. The investment and trade between China and Syria were seriously damaged by the Syrian War, which has continued since 2011. From 2003 to 2017, Syria’s exports to China dropped from 506,637.16 million dollars to 13,341.73 million dollars. From 2010 to 2017, China’s investment in Syria dropped from 8.12 million dollars to 0.53 million dollars, which led the geo-economic linkage intensity between China and Syria to decrease from 140.14 million dollars in 2006 to 18.96 million dollars in 2017. In particular, it is worth noting that the “Matthew effect” of China’s geo-economic linkage with MSRCs is significant. From 2006 to 2017, the geo-economic linkage intensities of Japan and South Korea increased by 77.80% and 215.17%, respectively, and the geo-economic linkage intensities of Sudan, Yemen, and Syria with China decreased by 17.57%, 41.57%, and 85.71%.

The geo-economic linkages between China and other countries in the region are far less intense than those with regional powers such as Japan, South Korea, India, and Saudi Arabia (Table 2). Taking Japan as an example, the intensity of China’s geo-economic linkage with Japan was far greater than that with nearby North Korea and South Korea from 2006 to 2017. In 2017, the intensity of China–Japan geo-economic flows reached 216,048.90 million dollars, accounting for 25.91% of the total intensity of geo-economic flows. In 2017, the intensity of China–Japan geo-economic flows reached 216,048.90 million dollars, accounting for 25.91% of the total intensity of geo-economic flows. The relative intensity of China and Japan’s geo-economic linkage has always been more than a quarter, far higher than those of other countries, although its proportion is declining, indicating that Japan’s geo-economic status is particularly important. From 2006 to 2017, the ratio of China–Singapore geo-economic linkage intensity to total geo-economic linkage intensity was greater than 5%, and the geo-economic linkage intensity between China and India was greater than those of Pakistan, Sri Lanka, and Bangladesh. In addition, the intensities of the geo-economic linkages between Saudi Arabia in Western Asia and Egypt in Africa and China were greater than those of other countries in the regions. From 2006 to 2017, the total intensities of the geo-economic linkages between China and Japan, Singapore, Saudi Arabia, India, and Egypt accounted for more than 38%, reaching 55.44% in 2006. These figures show that regional powers play an important role in the process of geo-economic cooperation between China and the MSRCs. In the future, geo-economic cooperation between China and the MSRCs should be oriented toward regional powers. It is worth noting that although relevant studies have emphasized that China’s geo-economic linkages have a geographical proximity effect [54], technological progress and transportation advances have made the impact of distance on geo-economic interaction weaker and weaker, and the geographical proximity effect of China’s geo-economic linkage with MSRCs is only significant in a few countries.

### 4.2. Analysis of Geo-Economic Relation Types between China and the MSRCs

The interactions of geo-economic flow and the differences in the elements of geo-economic flow cause the different types of geo-economic relations to show different characteristics. Geo-economic linkage intensity can reveal how close the relations between China and the MSRCs are, but it cannot reveal the core characteristics of the geo-economic relations. Therefore, we used the geo-economic linkage intensity as the explained variable, the geo-economic type indices as the explanatory variable, and extreme random forest regression to classify the types of geo-economic relations between China and the MSRCs.

#### 4.2.1. Index System of Geo-Economic Relation Types

Competition and cooperation are the most basic paradigms of geo-economic relations, and their deep-rooted influences cause the studies of types of geo-economic relations to rely on obvious cooperative and competitive tendencies. In earlier research, the logic of competition and cooperation gradually evolved into various forms, such as symbiosis, complementarity, competition, games, and hostility [55]. The geo-economic relations between countries are summarized as competition-dominated or cooperative-dominated, and the types of geo-economic relations are divided into competition and cooperation. However, regarding the interactions of geo-economic elements’ flow, geo-economic activities between countries always coexist with competition and cooperation. Complexity, diversification, dependence, and flow space are all characteristics of geo-economic relations. If the types of geo-economic relations are divided into competition and cooperation, this not only ignores the multiple attributes of geo-economic relations, but also ignores the roles of endogenous and exogenous elements, such as geographic basis, market power, and resource endowments, in geo-economic relations. In fact, the development of geo-economics is the result of a combination of various elements, such as geographic location, geopolitics, market demand, and complementary resource endowments. Proximity helps ensure a favorable geo-economic location and lays the foundation for the construction of border geo-economic cooperation zones. The geo-economic relations between countries depend to a certain extent on their geopolitical relations, and the international economic cooperation network and economic corridor construction built by the BRI have deepened the geo-economic cooperation between China and the MSRCs. In this era of informatization and globalization, market forces are stronger than ever, and trade flow and investment flow are the core flow elements that constitute the maintenance of geo-economic relations. The growth of international trade is driven by the growth of the global import and export markets, and the growth of investment flow is driven by the prosperity of the international financial market. The heterogeneity of resource endowments determine that countries need to allocate resources on a global scale and promote geo-economic competition or cooperation among countries so they occupy favorable geo-economic positions in the global production network. Therefore, the analysis of the types of geo-economic relations cannot be confined to the logic of competition and cooperation, but should extend to the dominant elements of geo-economic relations, such as distance, geopolitics, markets, and resources, in order to better understand the dominant characteristics of the types of geo-economic relations.

In fact, the dominant elements of geo-economic relations include not only distance, politics, market, and resources, but also geo-culture and industrial structure. However, these are not the standard for the classification of geo-relation types in this study, primarily for the following reasons. First, the East Asian civilization centered on China has limited influence on the Maritime Silk Road countries, and its scope mainly extends to Japan, South Korea, North Korea, and Southeast Asian countries, and thus geo-culture cannot be used as a basis for judging the types of geo-economic relations with the MSRCs. Second, the geo-economy is affected by industrial structure complementation and industrial transfer, but China’s current industrial structure adjustment and optimization are still continuing, resulting in a weak contribution to geo-economic development. In addition, relevant studies have shown that geo-economic relations are significantly affected by elements such as the market, geographic distance, and geopolitics [56]. China and the MSRCs have close cooperation in resources and energy, and the development of bilateral geo-economics is highly correlated with resource endowments. Therefore, this study classifies the types of geo-economic relations according to the four main geo-economic relations factors of distance, geopolitics, market, and resources, and establishes an index system for the types of geo-economic relations (Table 3).

The distance index usually includes the distance between the capitals and whether the borders are adjacent. On the other hand, the development of modern transportation technology makes transportation time an important index of the influence of geographic distance. Since the economic activities of China and the MSRCs mainly rely on shipping, we select the distance between capitals, whether there is a common boundary, and shipping time as the indices to measure the dominant factors of distance. The cooperative goldenstein factor and conflict goldenstein factor reveal the degrees of geopolitical cooperation and conflict between countries. In view of the important role of geo-economic cooperation documents between China and MSRCs, such as Belt and Road cooperation memorandums, trade agreements, investment agreements, and economic cooperation framework agreements, we take geo-economic cooperation documents as supplementary indexes of dominant geopolitical factors. By searching the treaty documents on the website of the Ministry of Foreign Affairs of the People’s Republic of China (https://www.mfa.gov.cn/web/ziliao_674904/tytj_674911/tyfg_674913/, accessed on 3 October 2022), we collect 110 Belt and Road cooperation memorandums, trade agreements, investment agreements, economic cooperation framework agreements, technical cooperation agreements, oil and gas cooperation agreements, and assistance agreements, which are signed by the government of the People’s Republic of China, the national development and reform commission of the People’s Republic of China, the Ministry of Foreign Affairs of the People’s Republic of China, the Ministry of Commerce of the People’s Republic of China, and MSRCs. According to the signing of geo-economic cooperation documents, we apply a Likert scale to give scores to geo-economic cooperation documents. The size of a country’s market is closely related to its GDP, per capita GDP, and population. The larger the GDP, the higher the GDP per capita, and the larger the population, the larger the country’s market size. The natural resource endowments of various countries vary greatly, and the spatial distributions of minerals and energy are even more heterogeneous. The total rent of natural resources is the sum of the rents of energy, minerals, and forest resources, reflecting the abundance of a country’s natural resource endowments. The exportation of fuel, ore, and metal reflects a country’s mineral exports. China is a major importer of oil and natural gas, and the oil and gas exported by a country to China can thus reflect the role of oil and gas resources in its geo-economic relations with China. Therefore, we select the fuel exports, total rents of natural resources,, ore and metal exports, and the value of oil and gas exported to China as indices to measure the dominant factors of resource.

#### 4.2.2. Measurement of Geo-Economic Relation Types

**Figure 4:**
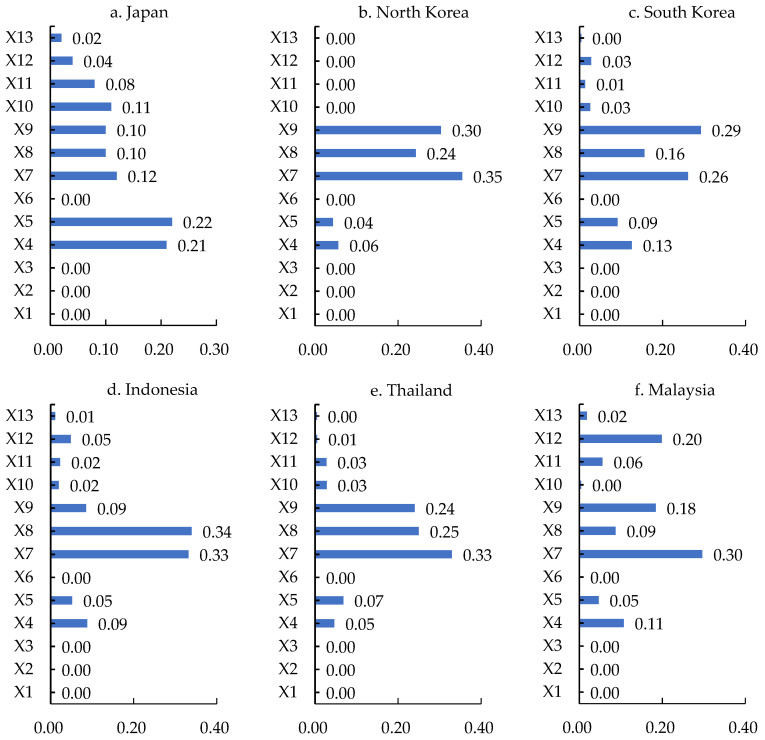

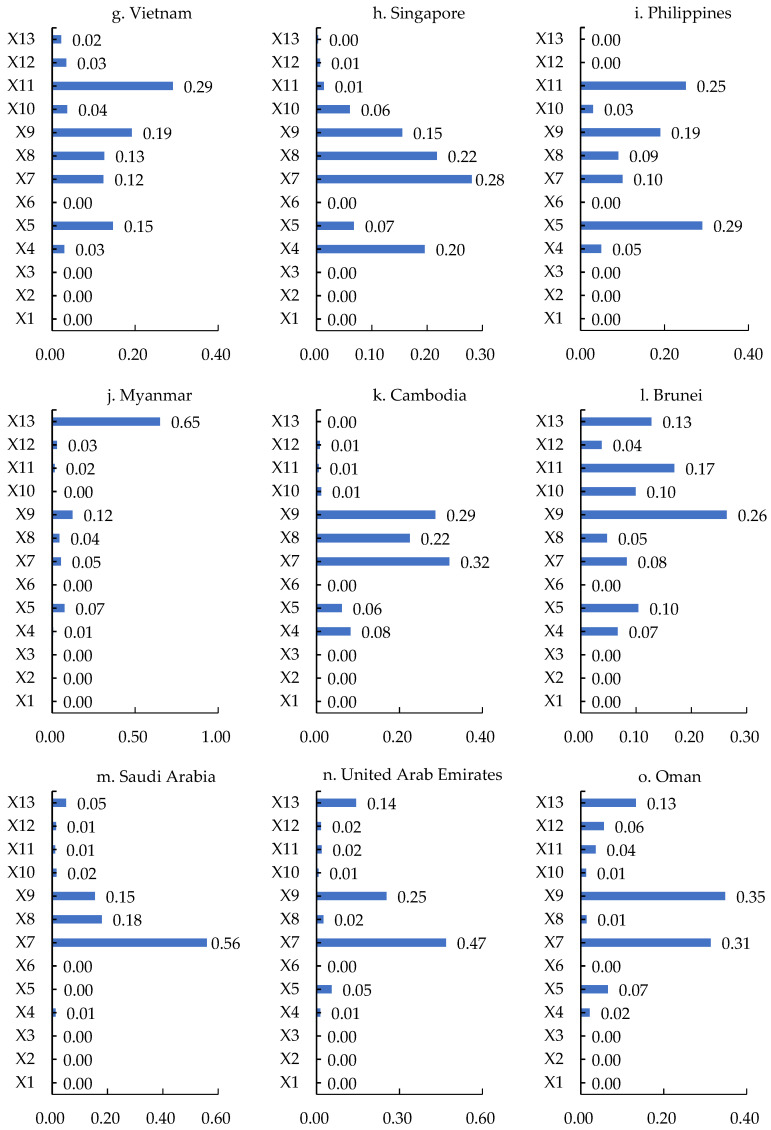

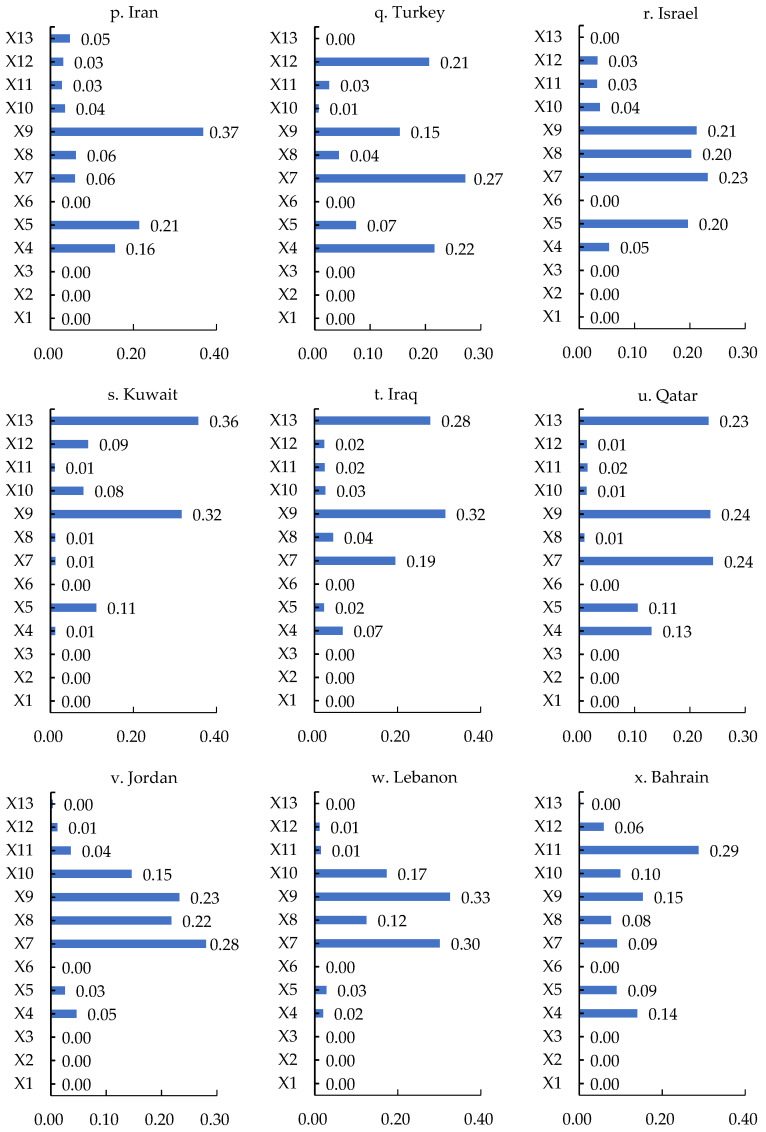

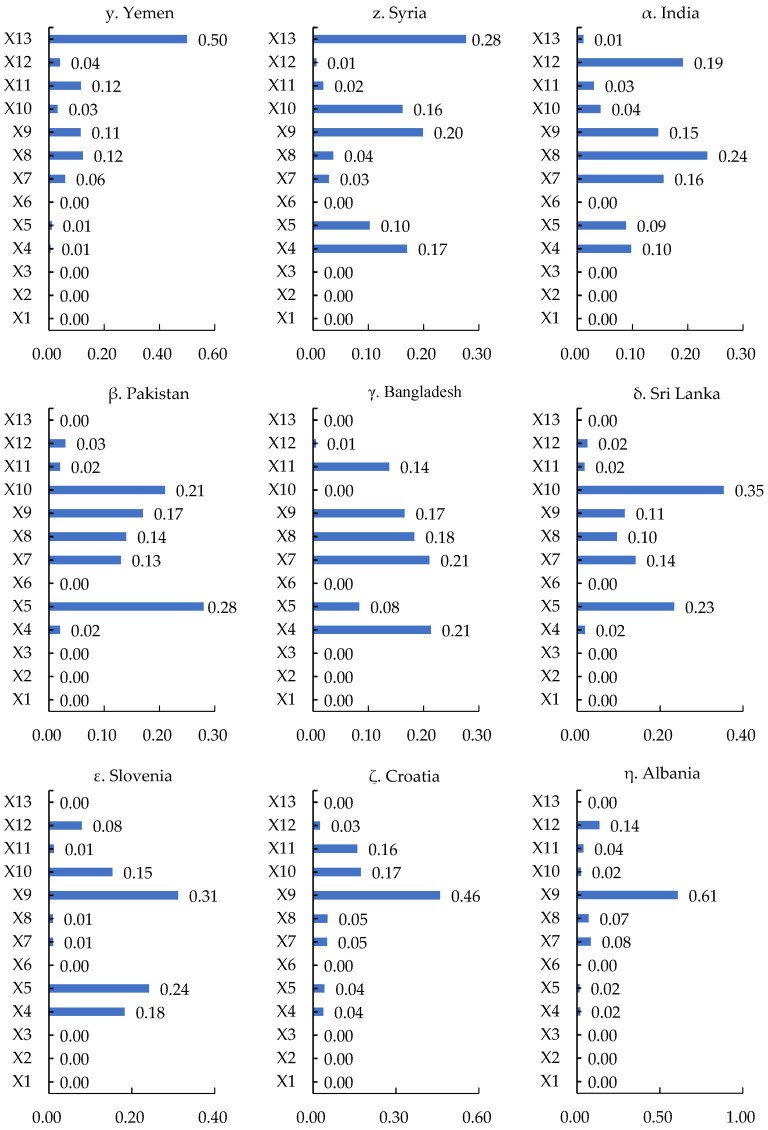

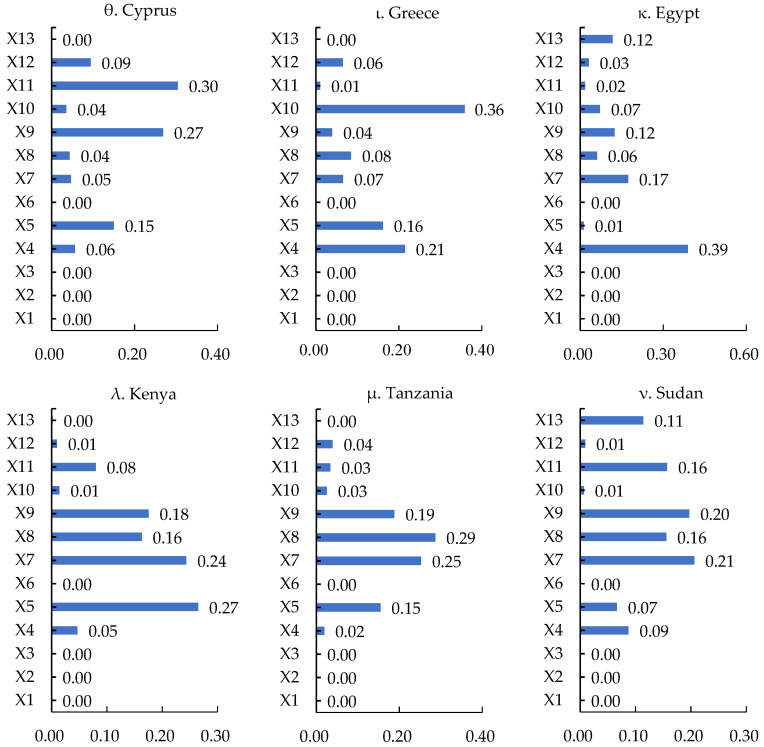
Type indices showing the characteristic importance of geo-economic relations between China and MSRCs. Note: The greater the value, the greater the importance of type indices.

Although there may be correlations between geo-economic relation type indices, extreme random forest regression is not sensitive to multivariate collinearity. Therefore, the correlation of geo-economic relation type indices will not affect the measurement of the importance of geo-economic relation type indices. According to the measurement of the importance of geo-economic relation type indices in MSRCs by extreme random forest regression, the results are shown in Figure 4.

According to the geo-economic relation type indices’ importance for China and the MSRCs, it can be seen that the dominant elements affecting the geo-economic relations between China and the MSRCs are significantly different (Figure 4). In terms of indices, the top indices of different countries are not uniform. GDP, per capita GDP, the value of oil and gas exported to China, the total rent of natural resources, and the cooperative goldenstein factor all appear as the most important indices. GDP and population are the most important characteristics overall of the geo-economic relations between China and the MSRCs. There are 12 countries with GDP as the most important, and 9 countries with population as the most important. This shows that national economy size and market are particularly important in geo-economic linkages, and these are the top indices for the development of geo-economics between China and many MSRCs. Other important indices that appear frequently are the cooperative goldenstein factor, the value of oil and gas exported to China, and the export of ore and metal, which means that geopolitical cooperation, ore resources, oil, and gas resources are of great significance to the geo-economic development of China and the MSRCs. The importance of each of the indices of the distance between the capitals of the countries, whether the boundary is adjacent, and shipping time is 0, which shows that distance has a very weak effect on the geo-economic relations between China and the MSRCs and is not a major obstacle to China’s geo-economic development. In terms of importance values, the maximum value of importance is 0.65, accounting for 65% of the importance of all indices in the country, indicating that this individual index plays a decisive role in the development of the geo-economy in a few countries and China. For example, oil and natural gas from Myanmar and Yemen are exported to China. The differences in geographic location, natural resource endowments, geopolitical relations, and market size among various countries have led to large fluctuations in the importance of the indexes in various countries. For example, the importance of the cooperative goldenstein factor varies greatly across countries, with that of the Philippines being 108 times that of Saudi Arabia (Figure 4).

The geopolitics indices, market indices, and resource indices play key roles. Among the types of indices affecting the geo-economic linkages between China and Japan, the Philippines, Kenya, and Pakistan, the cooperation goldenstein factor is the most important, whereas for China and Egypt, the conflict goldenstein factor is the most important. Due to the prominent position of geopolitics, the geo-economic relations between China and these countries are very sensitive to bilateral geopolitical exchanges. Among the types of indices that affect the geo-economic relations between China and North Korea, Thailand, Malaysia, Singapore, Cambodia, Saudi Arabia, the United Arab Emirates, Turkey, Israel, Qatar, Jordan, Bangladesh, and Sudan, the most important is GDP (Figure 4). For China and Brunei, South Korea, Oman, Iran, Iraq, Slovenia, Croatia, and Albania, population is the most important, whereas for China and Indonesia, India, and Tanzania, the most important is gross domestic product per capita (Figure 4). The market indices are powerful driving forces for China and these countries to develop geo-economically and form close geo-economic linkages in global trade, investment, and production networks. Among the types of indices affecting China’s geo-economic relations with Kuwait, Myanmar, Yemen, and Syria, the most important is the value of oil and gas exported to China (Figure 4). For China, as well as Sri Lanka and Greece, the most important is the percentage of fuel exports as a percentage of commodity exports. For China and Cyprus, Vietnam, and Bahrain, the most important is the total rental value of natural resources. These countries are rich in mineral, oil, and gas resources and agricultural products, and export large amounts of resource products to China, forming a stable flow of trade and resources and promoting sustainable bilateral geo-economic development.

To judge the validity of the extreme random forest regression model in measuring the importance levels of the geo-economic relation type indices, we used the R^2^, MSE, MAE and EVS to evaluate the estimation effect of the extreme stochastic forest regression model (Table 4). The R^2^ of the MSRCs is 1, so the extreme random forest regression model has a very good fit, and the importance of geo-economic relation types was measured very well. The MSE and MAE of the MSRCs are close to 0, which shows that the extreme random forest regression model has very small errors, the model estimation accuracy is very high, and its estimation effect is close to perfect. The EVS of the MSRCs is 1, and the interpretation strength of the geo-economic relation type indices for the geo-economic relation type reaches 100%, which demonstrates that the extreme random forest regression model is a good measure of the importance of the geo-economic relation type indices.

#### 4.2.3. Classification of Geo-Economic Relations between China and the MSRCs

Most of the relevant studies classify geo-economic relations as competitive type or cooperative type when classifying the types of geo-economic relations [57]. However, the classification of geo-economic relation types under the logic of competition and cooperation cannot reveal the key factors that dominate the geo-economic interaction between China and MSRCs. The analysis of the importance of the geo-economic relation type indices’ characteristics not only identifies the importance of each dominant element in geo-economic relations, but also lays the foundation for the classification of geo-economic relation types. The sum of importance of all types geo-economic relation type indices is 1. If the total importance of one index or several indices exceeds 0.5, then it is dominant. Therefore, based on the importance of individual distance, market, geopolitics, and resource indices exceeding 0.5, the geo-economic relations between China and MSRCs are divided into market-oriented (M) and resource-oriented (R). At the same time, according to the total importance, if the two dominant factors of market and resources, market and geopolitics, resources, or geopolitics exceed 0.5, the geo-economic relations between China and MSRCs are classified as market-resource-oriented (M-R), market-political-oriented (M-P), or resource-political-oriented (R-P).

**Figure 5:**
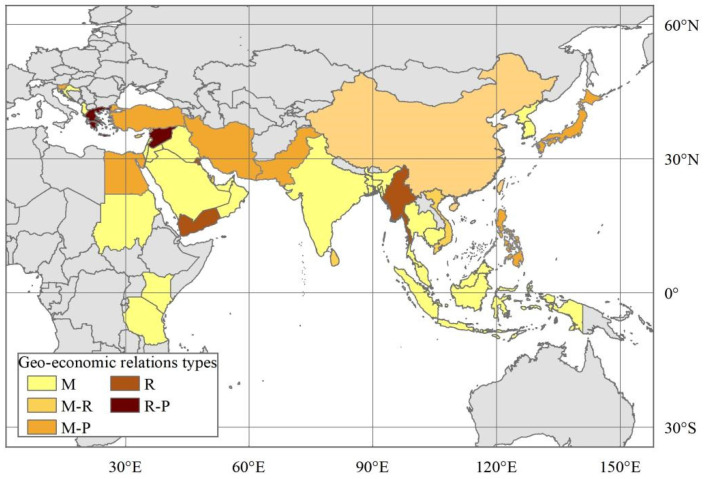
Types of geo-economic relations between China and the MSRCs. Note: The map is a drawing is based on the standard map of GS (2016) 1667 that comes from the standard map service system of the Ministry of Natural Resources of the People’s Republic of China (http://bzdt.ch.mnr.gov.cn/index.html, accessed on 3 October 2022).

Market-oriented (M) is the most important type of geo-economic relation between China and the MSRCs. Among the 39 MSRCs, 21 are market-oriented geo-economic relations, accounting for 53.85%. From a spatial point of view, the market-oriented type is distributed in East Asia, Southeast Asia, South Asia, West Asia, the Balkans, and Africa (Figure 5). South Korea and North Korea in East Asia are geographically adjacent to China, making investment and trade convenient. China and South Korea have developed economic and trade relations and are important import and export markets for each other, and the market indices’ importance between China and South Korea is as high as 0.71. Affected by North Korea’s national conditions, the close geopolitical relations between China and North Korea have not effectively driven the deepening of bilateral geo-economic cooperation, making the market indices’ scores much higher than those of the geopolitics indices, and the bilateral geo-economic linkages are oriented by the market. The importance levels of the market indices in Thailand, Cambodia, Malaysia, Singapore, and Indonesia are 0.82, 0.83, 0.57, 0.65, and 0.76, respectively. The construction of the China-ASEAN free trade area promotes the rapid development of bilateral geo-economics. China, Thailand, Cambodia, Malaysia, Singapore, and Indonesia have huge trade flows and investment flows, making them important geo-economic markets for each other. India and Bangladesh in South Asia have large populations, and the importance levels of the population for the two countries are 0.15 and 0.17 respectively. Moreover, the national economic aggregate and per capita GDP of India and Bangladesh are of greater importance, and the market indices dominates economic trade relations and investment relations with China. Saudi Arabia, the UAE, Oman, and Iraq are located in the Middle East and are rich in oil and gas resources. China imports large quantities of oil and gas resources and exports a large number of commodities to these countries, making the importance of the market indices more than 0.5. Israel has strong technological capabilities and has attracted a large amount of Chinese investment. China has become the largest source of imports and an important trading partner of Jordan and Lebanon, and Lebanon has become an important overseas market for China in the Middle East. Although Croatia and Albania in the Balkans are small, their imports and exports have grown rapidly, and they have become important export and investment markets for China in the Balkans. The importance levels of the market indices for Kenya, Tanzania, and Sudan are 0.58, 0.73, and 0.56, respectively. Kenya, Tanzania, and Sudan, as African node countries on the Maritime Silk Road, are rich in resources, large in population, and have huge potential markets. The vast investment markets of Kenya, Tanzania, and Sudan have attracted a large amount of Chinese investment, and they have become some of China’s most important markets in Africa. It is worth noting that the market-oriented type has formed obvious agglomerations in Southeast Asia and the Middle East, which further shows that the positioning of market-oriented geo-economic relations is significant.

Countries which are resource-oriented (R) are Yemen, Myanmar, and Kuwait (Figure 5). Kuwait is rich in oil and gas resources; oil exports account for 95% of its total exports. Its oil and gas exports to China occupy an important position in its economic and trade relations with China. In 2017, Kuwait exported 7083 million dollars of oil and gas to China, accounting for 79.27% of Kuwait’s total exports to China, which made the importance of its oil and gas value index for exports to China as high as 0.36, and the resource indices’ importance reached 0.54. Myanmar’s economic development level is low, and the exportation of resources such as timber, agricultural products, and mineral products is an important part of its geo-economic linkages with China. After the opening of the China–Myanmar oil and gas pipeline, a large amount of oil and gas was imported into China through Myanmar, and the importance of the oil and gas value index for exports to China reached 0.63. Therefore, oil and gas constitute an important part of the geo-economic linkages between China and Myanmar. Yemen is one of the least developed countries, and its economic development depends on the exportation of oil. Yemen’s exports to China are mainly crude oil, and the importance of the oil and gas exported to China is as high as 0.5. On the whole, countries whose geo-economic relations with China are resource-oriented are mainly resource-rich countries with backward economic development. Their geo-economic relations with China rely on advantageous resource exports, and the development of bilateral geo-economic relations is relatively weakly affected by the market, geopolitics, and distance.

The market-oriented and resource-oriented (M-R) relations are with Vietnam, Brunei, Qatar, Bahrain, Sri Lanka, and Cyprus (Figure 5). Vietnam has a young population structure and a strong consumption momentum. Its fast-growing market has promoted a continuous increase in trade between China and Vietnam. The importance levels of its GDP, per capita GDP, and population are 0.12, 0.13, and 0.19, respectively. On the other hand, Vietnam is rich in minerals and tropical agricultural products, and it exports large amounts of minerals and tropical agricultural products to China, and the importance of the resource indices is 0.38. The market and resources constitute the key to geo-economic growth between China and Vietnam. Brunei, Qatar, and Bahrain have relatively developed economies with high per capita national incomes and strong consumption capacity of their residents. Their markets have become key to attracting investment and promoting imports. At the same time, the export of oil and gas resources to China has become another key to China’s geo-economic linkages. The indices of market and resource importance are close to 0.8, indicating that these factors together dominate the geo-economic linkages. Sri Lanka’s economy is relatively backward, and resource products such as agricultural products, metals, minerals, and timber account for relatively high proportions of its exports to China. Among them, the importance of fuel exports as a percentage of merchandise export reach 0.35. China and Sri Lanka have close economic, trade, and investment cooperation. A large number of Chinese products are exported to Sri Lanka, and many Chinese companies invest in Sri Lanka, which has caused the market to become an important driving force for bilateral geo-economic development. Cyprus has a relatively developed economy, a high national income, rich copper mines, and tourism resources. China not only exports large amounts of commodities to Cyprus, but also imports its copper mineral products, causing the market and resources to serve as the basis of bilateral geo-economic development. On the one hand, countries with market-resource-oriented geo-economic relations rely on their rich resources and resource-based product exports to maintain close geo-economic linkages. On the other hand, they rely on the huge market brought about by a large population or high national income to promote trade and investment.

The geo-economic relations between China and Japan, the Philippines, Iran, Turkey, Pakistan, Egypt, and Slovenia are dominated by market-political-oriented (M-P) means. These countries have each built a relatively complete market economy system and have a close market linkage with China. Among these, Japan and Slovenia have developed economies and high consumption levels, whereas the Philippines, Iran, Turkey, Pakistan, and Egypt have larger populations and larger markets. The importance levels of the conflict goldenstein factor in Japan, the Philippines, and Turkey are 0.21, 0.05, and 0.22, respectively. Geopolical events such as the China–Japan Diaoyu Islands dispute, the China–Philippines Huangyan Island dispute, and Turkey’s support for "East Turkistan" terrorists seriously affected their geo-economic interactions with China. China has close geopolitical relations with Pakistan, Iran, Egypt, and Slovenia, and geopolitical cooperation has greatly promoted bilateral geo-economic linkages. China and Pakistan are “iron brothers” with close geopolitical relations, and the China–Pakistan Economic Corridor has become a model of geo-economic cooperation under the framework of the Belt and Road. China and Iran have established a comprehensive strategic partnership, and political mutual trust has promoted the continuous deepening of cooperation between the two countries in various fields, such as energy, trade, and transportation. As the fulcrum country of the BRI in Africa, Egypt has linked its development strategic plan with the construction of the Belt and Road, which promoted the joint construction of the Suez Economic and Trade Cooperation Zone and the New Administrative Capital. Slovenia has responded actively to the BRI, as a result of which China and Slovenia have signed a number of cooperation documents, such as a memorandum of understanding on jointly building the BRI, which has promoted the continuous growth of bilateral economic and trade investment cooperation. It is worth noting that the market is more sensitive to geopolitics, the market-politics-oriented type of geo-economic relations have significant fragility, and geopolitical conflicts can easily lead to a worsening of such geo-economic relations.

Only Syria and Greece are resource-dominant and politically dominant (R-P) countries (Figure 5). Syria is rich in oil and gas, and before it fell into a civil war, China and Syria had close energy cooperation. Large amounts of Syrian oil and gas are exported to China, and the importance of the oil and gas exported to China as Syrian exports is 0.28, ranking first among all important indices in Syria. China and Syria still have close political relations, and the Chinese government and companies have actively helped Syria restore its economic damage from the war. Greece is rich in mineral and forest resources. Resource-intensive products such as stone, fossil fuel, pulp, and paperboard are exported to China in large quantities, and trade with China has grown steadily. The political relations between China and Greece are stable; both sides have accumulated solid mutual trust in their long-term exchanges; and the geopolitical index has reached 0.37 in importance. As a hub country for the Belt and Road construction in Europe, Greece regards the Belt and Road as a development opportunity and actively promotes strategic alignment and upgrades cooperation in the fields of infrastructure, energy, and transportation. Advantageous resources have always been one of the key elements of geo-economic linkages as well as an important factor in geopolitical rivalry. When resources and geopolitics are combined to shape geo-economic relations, they will be more stable and reliable.

On the whole, the market-oriented type is the most important type of geo-economic relation between China and the MSRCs, and the market-politics-oriented type is the second-most-important type of geo-economic relation. The market-resource-oriented, resource-oriented, and resource- and politics-oriented types are scattered. In the era of geo-economics in the context of globalization, the market economic system has long become the most important economic system in the world. The geo-economic activities of various countries are no longer restricted to the scope of geopolitics, but activities of trade, investment, and geo-economics are carried out under the influence of the market. The multinational operation of enterprises is oriented toward seeking market profits, and geo-economic contacts among countries take economic benefits as an important goal. In geo-economic activities, the position and power of the market are stronger than ever before, making it the dominant force in geo-economic linkages. The nation is the most important subject of geo-economic relations, which determines that geopolitical relations will inevitably affect geo-economic activities. In the geo-economic ties between China and the MSRCs, overseas aid projects, infrastructure connectivity, and economic corridor construction promoted by the country as the mainstays have become important ways to deepen geo-economic linkages. At the same time, major geopolitical conflicts have led to serious setbacks in the geo-economic relations between China and countries involved in geopolitical conflicts. Resource-rich countries have the resources needed for the economic development of other countries, and exporting superior resources is conducive to promoting geo-economic linkages. When resource exports become the most important way for a country to develop its geo-economy, it becomes the main force shaping the country’s geo-economic relations. For economically underdeveloped countries, exporting superior resources or developing resources is the backbone of their national economy, and their external geo-economic relations depend to a large extent on the export of resources. Obviously, the cross-border flow of resources is of great significance to the geo-economic linkages of some countries. The market, resources, and politics do not work in isolation, but also work together to encourage markets, resources, and politics to jointly maintain geo-economic relations.

## 5. Conclusions and Policy Implications

In the context of geo-economic globalization and the reality of major changes unseen in a century, maintaining and handling the geo-economic relations between China and the countries along the Belt and Road are China’s realistic needs. This paper analyzed the types of geo-economic relations between China and the MSRCs, and found that the geo-economic linkage intensity between China and the MSRCs has continued to increase, whose growth phase characteristics are significant. The unbalanced development of investment and trade between China and MSRCs hinders the enhancement of the strength of bilateral geo-economic linkages. China has strong geo-economic linkages with Japan, South Korea, and nine Southeast Asian countries; and regional powers generally have an important geo-economic status. The “Matthew effect” of China’s geo-economic linkage with MSRCs is significant. The classification of geo-economic relation types should go beyond the logic of competition and cooperation and analyze the multiple attributes of geo-economic relations with various influential factors, such as geographic location, geopolitics, market demand, and resource endowments. There are obvious differences in the dominant factors affecting the types of geo-economic relations between China and MSRCs, and the distribution of the importance of the indices of the types of geo-economic relations in each country is disordered. Distance plays a very weak role in the geo-economic relations between China and the MSRCs, but plays an important role in geopolitics, markets, and resources. There are five types of geo-economic relations between China and the MSRCs: market-oriented, resource-oriented, market-resource-oriented, market-geopolitics-oriented, and resource-geopolitics-oriented, of which market-oriented is the most important type of geo-economic relations.

It is of great guiding significance to analyze the characteristics of geo-economic linkages and types of geo-economic relations between China and MSRCs for promoting the geo-economic cooperation between China and MSRCs. In order to further promote the development of geo-economic relations between China and MSRCs, China should adopt the following important policies.

(1) China should focus on regional powers along the Maritime Silk Road in bilateral geo-economic cooperation. The importance of MSRCs to China’s geo-economic development is not indiscriminate, and regional powers have a greater say in regional geo-economic affairs. Regional powers such as Japan, South Korea, and India occupy important positions in the regional geo-economic development; and Singapore, Pakistan, Egypt, and other countries are the hubs of the shipping channels of the Maritime Silk Road, which are of great significance to China’s construction of the 21st-Century Maritime Silk Road. Therefore, China should be guided by regional powers in its bilateral geo-economic cooperation with MSRCs, maximize the advantage of its economic scale, and continue to promote the steady growth of trade and investment between itself and the regional powers along the Maritime Silk Road. At the same time, China should improve the geo-economic cooperation mechanisms and strengthen political and economic cooperation with countries along the Maritime Silk Road as transportation hubs.
(2) Strengthening geopolitical cooperation with MSRCs. The importance of geo-economic relation type indices indicates that geopolitics indices have a strong effect on geo-economic relations. Although the construction of the 21st-Century Maritime Silk Road has been actively supported by many countries, regional powers such as Japan, India, and Turkey have not signed an inter-governmental document on jointly building the Belt and Road with China, and suspicion and vigilance about the construction of China’s Maritime Silk Road still continue. Therefore, China should strengthen geopolitical exchanges with MSRC, reduce or eliminate geopolitical suspicions, and strengthen geopolitical cooperation.
(3) Formulating cooperation plans according to the types of geo-economic relations. Due to the differences in geo-economic elements, there are different types of geo-economic relations between China and MSRC, and the cooperation demands of different geo-economic relation types are different. In deepening geo-economic cooperation with MSRC, China should formulate cooperation plans according to geo-economic relation types, take into account the characteristics of the geo-economic development of the cooperative countries, improve the degree of compatibility of geo-economic cooperation between the two sides, and promote potential geo-economic cooperation in practice.

In the era led by the “flow space” theory of geo-economics, geo-economic relations have obvious attributes of flow. The interactions of geo-economic flows and the differences in their dominant elements lead different types of geo-economic relations to show different characteristics. Compared with related studies that emphasize the competition and cooperation of geo-economic relations [57], we believe that geo-economic relations have various attributes, and the analysis of the types of geo-economic relations between China and MSRCs countries should return to the dominant factors of geo-economic relations, such as distance, geopolitics, markets, and resources. We used the flow of geo-economic elements and dominant factors to identify and classify the types of geo-economic relations, allowing us to scientifically identify the types of geo-economic competition and cooperation between countries, as well as to identify key elements in geo-economic cooperation between countries more accurately, and lay the foundation for the formulation of geo-economic strategies. The BRI promotes inclusive globalization beyond the conflicting logic advocated by traditional geo-economic theory [3], and the Belt and Road should no longer be completely confined to the paradigm of competition and cooperation, but should return to the geo-economic relations themselves to understand the multiple characteristics of geo-economic relations. The analysis of the types of geo-economic relations is a complex project that should be continuously promoted. In the future, research on the types of geo-economic relations should pay more attention to the differences between different types of geo-economic relations, the formation mechanisms of various types of geo-economic relations, and the matching of geo-economic strategies and types of geo-economic relations.

## Figures and Tables

**Table 1:** MSRCs and regional divisions.

Regional	Countries
three East Asian countries	Japan, South Korea, North Korea
nine Southeast Asia countries	Indonesia, Thailand, Malaysia, Vietnam, Singapore, Philippines, Myanmar, Cambodia, Brunei
four South Asian countries	India, Bangladesh, Pakistan, Sri Lanka
fourteen West Asia countries	Saudi Arabia, United Arab Emirates, Oman, Iran, Turkey, Israel, Kuwait, Iraq, Qatar, Jordan, Lebanon, Bahrain, Yemen, Syria
five Balkans countries	Greece, Slovenia, Croatia, Albania, Cyprus
four African countries	Egypt, Kenya, Tanzania, Sudan

**Table 2:** The variation in the geo-economic linkage intensity of regional powers (million dollars).

Country	2006	2008	2010	2012	2014	2016	2017
Japan	121,513.73	171,436.02	194,132.62	184,474.52	208,243.62	201,161.28	216,048.90
Proportion of Japan	42.96%	39.52%	37.29%	29.78%	27.49%	27.33%	25.91%
South Korea	66,582.57	100,299.76	120,787.34	146,936.85	200,639.03	183,689.56	209,850.48
Proportion of South Korea	23.54%	23.12%	23.20%	23.72%	26.49%	24.96%	25.17%
Malaysia	15,640.65	24,219.31	32,595.01	48,803.47	74,553.03	62,286.53	59,824.88
Proportion of Malaysia	5.53%	5.58%	6.26%	7.88%	9.84%	8.46%	7.17%
Singapore	18,594.64	27,143.75	31,435.97	38,490.34	45,284.99	45,990.28	52,755.06
Proportion of Singapore	6.57%	6.26%	6.04%	6.21%	5.98%	6.25%	6.33%
India	9264.93	20,653.39	24,371.74	25,780.00	25,683.86	19,007.20	29,906.09
Proportion of India	3.28%	4.76%	4.68%	4.16%	3.39%	2.58%	3.59%
Saudi Arabia	6795.62	14,772.61	15,010.70	27,422.44	26,419.75	20,672.96	23,199.67
Proportion of Saudi Arabia	2.40%	3.41%	2.88%	4.43%	3.49%	2.81%	2.78%
Egypt	635.56	1228.17	1852.24	2778.66	2961.26	2416.36	3111.35
Proportion of Egypt	0.22%	0.28%	0.36%	0.45%	0.39%	0.33%	0.37%

**Table 3:** Index system of geo-economic relation types.

Dominant fFactors	Index	Unit	Variable Name
Distance	Distance between capitals Common boundary (yes/no) Shipping time (days)	Km / Day	X1 X2 X3
Geopolitics	Conflict goldenstein factor Cooperative goldenstein factor Geo-economic cooperation documents	/ / /	X4 X5 X6
Market	GDP GDP per capita Population	$ $/	X7 X8 X9
Resource	Fuel exports as a percentage of merchandise exports Total rent of natural resources as a percentage of GDP Ore and metal exports as a percentage of merchandise exports Value of oil and gas exported to China	% % % $	X10 X11 X12 X13

**Table 4:** Evaluation of the effect of the extreme random forest regression model.

Country	R^2^	MSE	MAE	EVS
Japan	1	0.00000000126033000	0.010369	1
North Korea	1	0.00000000000002964	0.000038	1
South Korea	1	0.00000000071889800	0.007218	1
Indonesia	1	0.00000000001375170	0.000949	1
Thailand	1	0.00000000001528910	0.000982	1
Malaysia	1	0.00000000005340960	0.002030	1
Vietnam	1	0.00000000002046850	0.000114	1
Singapore	1	0.00000000003645810	0.000169	1
Philippines	1	0.00000000000366551	0.000045	1
Myanmar	1	0.00000000000025831	0.000111	1
Cambodia	1	0.00000000000001940	0.000030	1
Brunei	1	0.00000000000000191	0.000012	1
Saudi Arabia	1	0.00000000001467870	0.000099	1
United Arab Emirates	1	0.00000000000782794	0.000667	1
Oman	1	0.00000000000059885	0.000199	1
Iran	1	0.00000000000457951	0.000568	1
Turkey	1	0.00000000000114745	0.000272	1
Israel	1	0.00000000000044109	0.000175	1
Kuwait	1	0.00000000000023910	0.000132	1
Iraq	1	0.00000000000054151	0.000187	1
Qatar	1	0.00000000000009229	0.000008	1
Jordan	1	0.00000000000001364	0.000029	1
Lebanon	1	0.00000000000001485	0.000031	1
Bahrain	1	0.00000000000000068	0.000007	1
Yemen	1	0.00000000000002779	0.000045	1
Syria	1	0.00000000000000332	0.000015	1
India	1	0.00000000001486080	0.001102	1
Pakistan	1	0.00000000000070024	0.000228	1
Bangladesh	1	0.00000000000049083	0.000159	1
Sri Lanka	1	0.00000000000010705	0.000082	1
Slovenia	1	0.00000000000000080	0.000023	1
Croatia	1	0.00000000000000330	0.000017	1
Albania	1	0.00000000000000003	0.000004	1
Cyprus	1	0.00000000000000219	0.000013	1
Greece	1	0.00000000000006996	0.000075	1
Egypt	1	0.00000000000031988	0.000147	1
Kenya	1	0.00000000000000610	0.000006	1
Tanzania	1	0.00000000000001063	0.000003	1
Sudan	1	0.00000000000001721	0.000035	1

## Data Availability

The datasets used and analyzed in the current study are available from the corresponding author on reasonable request.
